# Topical Skin Treatment and Its Influence on Surgical Healing: Review of Literature and Underlying Physiology

**DOI:** 10.1093/asjof/ojab029

**Published:** 2021-08-07

**Authors:** Alan D Widgerow, Mary E Ziegler, Laurie A Casas

**Affiliations:** Alastin Skincare, Carlsbad, CA, USA; Center for Tissue Engineering, University of California Irvine, Irvine, CA, USA; Division of Plastic and Reconstructive Surgery, The University of Chicago Medicine and Biological Sciences, Glenview, IL, USA

## Abstract

TriHex Technology (Alastin Skincare, Carlsbad, CA) has been shown clinically to promote healing and outcomes post procedures and has been demonstrated clinically to improve lipid droplet dissolution and patient-reported outcomes post procedure. Histologically, the formulations have proven to regenerate collagen and elastin. The use of the technology to prepare the skin for surgical procedures combined with its use post procedure was assessed through clinical study outcomes, histological evidence, and gene expression analyses and demonstrated remodeling of the extracellular matrix (ECM), accelerating healing, and initiation of anti-inflammatory genes. While the improvement in clinical signs and outcomes has been validated, the changes taking place at a molecular level need to be explored. The interaction of cells (adipocytes, macrophages, fibroblasts) and the ECM proteins (collagen, elastin) secondary to the effects of the topical agent application are discussed. It appears that the manipulation of fat during body contouring surgery and the resultant adipocytolysis precipitates a molecular profile that can be positively directed toward hastened healing by using adjuvant topical applications as preconditioning prior to surgery and after the surgical procedure. Here, we review the literature and underlying physiology relating to these products and describe how interleukin 6 appears to be the primary facilitator of these effects.

Surgical procedures disrupt cellular and extracellular structures that result in alterations in the hemodynamic, metabolic, and immune responses in the postoperative period that may manifest as changes in the skin. In general terms, the initial proinflammatory immune response is mediated by cells of the innate immune system, followed by a compensatory anti-inflammatory reaction mediated by the adaptive immune system.^1^ Macrophages and monocytes initiate proinflammatory cytokine production in the intraoperative and early postoperative periods at the initial injury site as part of the acute phase response.^1^

In addition, surgical procedures, by their nature, injure adipose tissue, leading to adipocytolysis, which initiates a change in the adipose phenotype to one of increased inflammation and decreased adipose-derived hormone expression (leptin, adiponectin). This response may be modulated by nutritional status, in particular preoperative dietary preparations.^2^ In this vein, it is interesting to speculate that through similar mechanisms, presurgical (and postsurgical) skin preparation may affect this acute surgical stress response.

Surgery also produces “waste product” accumulation as a by-product of the intervention. An aged or non-prepared extracellular matrix (ECM) may have impaired cellular recycling mechanisms involving the proteasome and autophagic processes.^3-5^ The utilization of topical agents enhances cellular recycling to improve outcomes (Figure 1).

**Figure 1.**
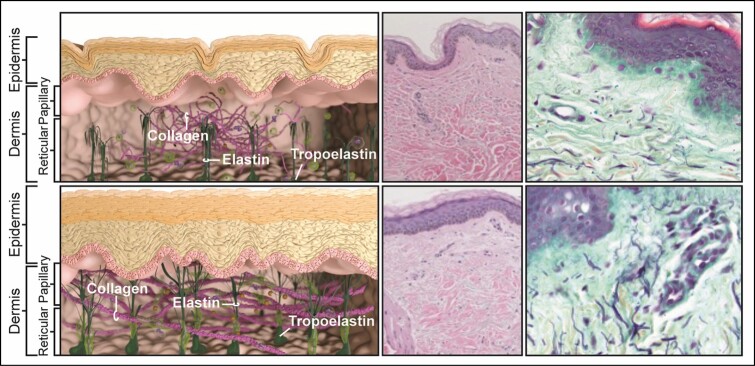
Topical agents improve outcomes by improving cellular recycling to generate healthy ECM. Top: Aged ECM—schematic left—(photodamage and intrinsic aging) results in thinned epidermis, clumped fragmented collagen, collapsed elastin—histology (H&E, top middle) shows thinned epidermis mature clumped collagen, flat basal layer (Movat right top)—sparse elastin especially in papillary dermis. Bottom: “Recycled” ECM with thickened epidermis, long intact collagen fibers, elastin “candelabra” fibrillin fibers extending to dermoepidermal junction—histology (H&E bottom)—thickened epidermis new freshened collagen, healthy basal layer (Movat right bottom)—new healthy elastin fibers in papillary and reticular dermis. ECM, extracellular matrix; H&E, hematoxylin and eosin.

Added to this, in surgical body contouring procedures, as fatty tissue and cells are destroyed through adipocytolysis, lipid droplets and free fatty acids (FFAs) are released from the adipose cells. These droplets and FFAs can be highly inflammatory, creating localized pockets of “inflammasomes” that can present as skin induration, hardened fibrous banding, and even fat necrosis where phagocytic and autophagic processes are overwhelmed.^4,6^ The FFAs induce inflammation by signaling macrophages and other immune cells to the area. Over time, this inflammation increases ECM protein production, leading to interstitial fibrosis in adipose tissue.^7^

By virtue of its very nature, most surgical procedures, especially those involving body contouring surgery, elicit adipocytolysis, characterized by inflammation arising from the disruption of adipocytes by interfering with lipolytic signaling pathways through the direct action of proinflammatory cytokines or catecholamines (Figure 2).^8^ Cell death by necrosis results in the release of intracellular molecules not found physiologically in the extracellular environment. In the case of fat cells, the released saturated fatty acids present in their lipid content act as endogenous molecules known as damage-associated molecular patterns (DAMPs), capable of activating toll-like receptor (TLR)-induced macrophage activation, leading to the production and liberation of proinflammatory cytokines, such as TNF-α (tumor necrosis factor alpha). In this proinflammatory setting, the activated macrophages polarize and become M1 macrophages that produce TNF-α, interleukin 6 (IL-6), nitric oxide (NO), among other proinflammatory molecules.^8^ IL-6 primarily regulates the hepatic component of the acute phase response resulting in the generation of acute phase proteins, including C-reactive protein (CRP). CRP levels increase approximately 4-12 hours after surgery and peak at 24-72 hours. Subsequently, CRP levels remain elevated for approximately 2 weeks.^1^

**Figure 2.**
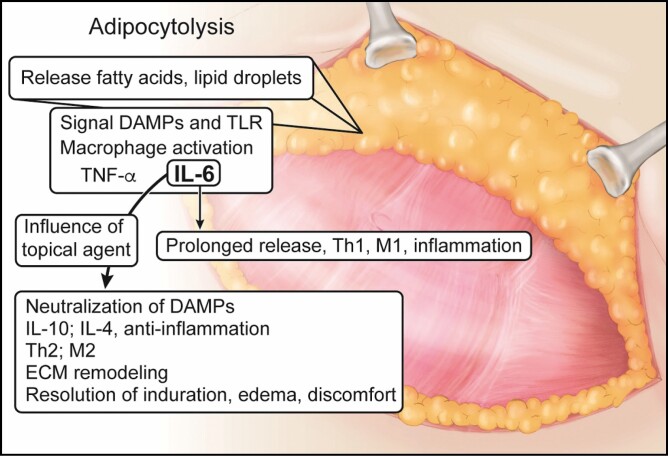
Regulation of adipocytolysis. Manipulation of fat tissue results in adipocytolysis and initiates DAMP and TLR signaling with the release of inflammatory mediators. The topical agents, through IL-6, neutralize the inflammation by initiating an anti-inflammatory reponse, resulting in the resolution of induration and edema. DAMP, damage-associated molecular pattern; IL-10, interleukin 10; IL-4 interleukin 4; IL-6, interleukin 6; M1, macrophage 1 cells; M2, macrophage 2 cells; Th1, T helper cells 1; Th2, T helper cells 2; TLR, toll-like receptors; TNF-α, tumor necrosis factor alpha.

This interaction occurring between lysed fat cells and macrophage activation is opposed by the neutralization of DAMPs and the anti-inflammatory cytokines produced and released by the adipose tissue.^8^ Adipose tissue macrophages are essential for the initiation and regulation of the inflammatory process. The classical macrophage activation, with proinflammatory M1 polarization, is responsible for mediating Th1-type immune responses. During properly regulated inflammation, this is followed by anti-inflammatory M2 polarization, also called alternative macrophage activation. In adipocytolysis, M1 macrophages are concentrated around the necrotic cells due to the local gradient of proinflammatory cytokines, while M2 macrophages are randomly dispersed by the adipose tissue.^8^ The local inflammatory consequences result in macrophages filled with triacylglycerol, known as foam cells, granulomatous panniculitis, and a high lipid efflux derived from the adipose tissue with consequent drainage through the lymphatic system. However, there is no evidence of systemic changes in the inflammatory profile of the post-procedure blood.^8^

Once the acute inflammatory stimulus is complete, the Th1-type immune response clears the acute inflammatory process. M1 macrophages reduce the production of proinflammatory cytokines and begin to produce anti-inflammatory cytokines as the macrophages revert to M2 polarization, which leads to immunomodulation and tissue repair. During this phase, the production of anti-inflammatory cytokines, such as IL-10 and IL-4, and growth factors, such as platelet-derived growth factor (PDGF), transforming growth factor beta (TGF-β), basic fibroblast growth factor (bFGF), and vascular endothelial growth factor (VEGF), set the stage for healing and remodeling. M2 macrophages also produce arginase, an enzyme that inhibits the production of free radicals present during M1 polarization. This anti-inflammatory effect is achieved by modulating TNF-α, generating increased IL-10 levels in the blood. In addition, M2 macrophages activate the acquired Th2 immune response, which completes the clearance of the remaining acute inflammation products and stimulates fibroblasts to release growth factors that induce ECM synthesis.^8^ In addition, although IL-6 and prostaglandin E2 function as proinflammatory cytokines in the early postoperative periods, they can also exert anti-inflammatory effects by attenuating TNF-α and IL-1 activity. Also, through its central role in the acute phase response, IL-6 induces macrophages to release prostaglandin E2, a powerful endogenous immunosuppressant and anti-inflammatory mediator. Prostaglandin E2 also stimulates the release of the potent anti-inflammatory cytokine IL-10. These profound anti-inflammatory effects result in a dramatic cytokine imbalance that is clinically referred to as the compensatory anti-inflammatory response syndrome. The initial proinflammatory phase of the host response to injury during the early postoperative period is followed by anti-inflammatory cytokine production by Th2-type lymphocytes, including IL-10, approximately 10 days after surgery. This is part of the immune modulation that restores depressed immune responses as well as downregulates hyperinflammation.^1^

## MODULATING RESPONSES TO SURGERY THROUGH THE SKIN

**Figure 3.**
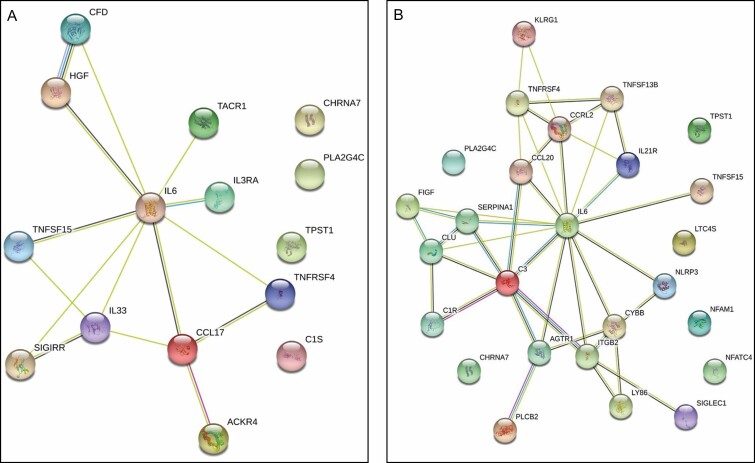
Protein-protein interaction networks for the 2-week groups. The String database was used to compare the interaction networks for the (A) 2-week untreated group and the (B) 2-week treated group. Reproduced by permission of Oxford University Press on behalf of The Aesthetic Society.^10^

In an effort to more effectively deal with the molecular by-products created by surgery and to optimize their elimination, certain peptides and active agents have been incorporated into topical preparations (TriHex Technology) to improve autophagic processes and macrophage efficiency.^9^ The mechanism of action of these agents has been tested through in vitro assays and clinical studies, as elucidated in previous publications.^1-4^ In particular and with reference to the physiological changes described in the background section above, the gene expression changes were monitored in patients undergoing liposuction in a split body comparison to compare the effect of topical applications (bland moisturizer—untreated vs active experimental product—treated) to the skin before and after surgery.^10-12^ Pretreatment was undertaken with different products (control moisturizing agent one side TriHex technology other) for 2 weeks prior to surgery in accordance with multiple protocols in the published clinical trials.^11-13^ This preconditioning timing of the TriHex technology is based on research done in relation to the time taken for the enzyme released by tripeptide-1 to begin clearing of fragmented collagen and elastin and replacement of new collagen and elastin as evidenced histologically.^5,14^ Patients were then treated for 10-week postsurgery comparing the resolution of skin changes. The liposuction was carried out on medial thighs with a volume that was consistent at 250 mL per medial thigh with similar suction techniques by 1 surgeon. The inflammatory gene expression profiles of the skin biopsy samples were evaluated from patients before and after liposuction from areas of the skin that were treated with different topical preparations. Biopsies were taken from the pretreated skin and the treated and untreated areas at 2 and 4 weeks after the procedure. All the gene expression comparisons were made relative to the pretreatment biopsy. The differential gene expression changes were predominantly upregulated among the groups (Figure 3).^10^

As anticipated, based on the physiologic response described in the background section above, IL-6 was the predominant mediator for the untreated and treated groups, with secondary reactions dependent on its initial release. However, the expression of IL-6 in the treated biopsies at 2 weeks was 1.7-fold higher than in the untreated group. IL-6 is a pleiotropic regulator of inflammation and immunity.^15^ Its expression initially functions as a warning signal to the entire body in the event of tissue damage.^16^ When examining the interaction network for each group, on the untreated side, the interaction network with IL-6 included CCL17, IL-33, and IL3RA. Bioinformatics analyses of these gene interactions suggested that the 2-week untreated group displayed a gene pattern that followed a typical inflammatory cascade after surgery, with an enhanced proinflammatory signal.^10^

**Figure 4.**
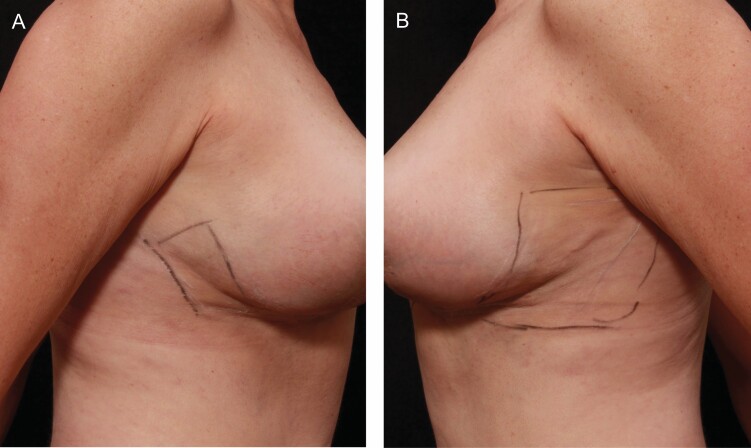
This 56-year-old female underwent bilateral breast reduction and axillary liposuction in split body trial. Comparison of investigator assessed induration in preconditioned right breast (A) compared with non-preconditioned breast (B) areas of induration, edema believed to correspond with inflammasomes prior to resolution.

In contrast, on the 2-week treated side, IL-6 showed interactions with IL21R, CCL20, CCRL2, and C3. Bioinformatics using STRING (https://string-db.org/), Gene Ontology (GO) and Reactome analyses revealed that the treated group shifted towards an immunomodulatory pattern, likely indicating an anti-inflammatory and M2 macrophage activation profile, which altered the proinflammatory cascade that was prominent in the untreated group. Moreover, the IL-6 interactions in the 2-week treated group were more extensive than those in the untreated group, with additional interactions with NLRP3 and CYBB. NLRP3 is an intracellular sensor that is a component of the NLRP3 inflammasome.^17^ Increased NLRP3 expression suggests a quickened inflammatory and wound healing response. Wounds lacking NLRP3 exhibit reduced growth factor and macrophage infiltration.^18^ There is also a link between the activation of autophagy and NLRP3. Inflammasome activation triggers autophagy induction, and autophagy eliminates activated inflammasomes, which are important for immune homeostasis and resolution of inflammation and induration (Figure 4A, B).^19^

The surgical procedure the patients in this study underwent results in cellular debris that must be cleared for proper healing. Part of this debris includes lipid droplets. These large particles, which are released by adipocytes, are digested through autophagy (lipophagy), which reduces these large lipid droplets into smaller products that macrophages can take up, thus providing clearance.^6,10,20^ The treatment used in this study is designed to stimulate macrophage clearance, offering a more efficient removal of lipid droplets, allowing for remodeling of the ECM.^14^ The gene expression patterns that emerged after the treatment clearly showed that at 2 weeks, the treatment enhanced the initial immune response with an M1 macrophage stimulation and the transformation to an anti-inflammatory response, and at 4 weeks, the treatment, as described below, finalizes the story by stimulating ECM remodeling.

Among the set of genes interrogated, only 2 were significantly upregulated in the 4-week untreated group: *CHRNA7* and *IL-11*. Thus, the overall gene expression profile of this group appeared to have returned to baseline. In contrast, the 4-week treated group showed a unique pattern of upregulation, with the disappearance of most of the significantly upregulated genes at 2 weeks, including *NLRP3*. The long-term overexpression of *NLRP3* is associated with chronic, nonhealing wounds, possibly due to the prevention of M2 macrophage polarization.^21,22^ A “switch” from M1-type macrophage to M2 inflammation resolution was suggested by these changes and is associated with regeneration and properly healing wounds. Taken together, the 4-week treated group revealed a gene pattern that showed that proinflammation was diminishing, and anti-inflammation was in full force.^10^ The 4-week treated group also showed an upregulation of *MMP25* and *TGM2*, indicative of ECM remodeling,^10,23^ which may explain the clinically observed decrease in fibrosis and/or skin hardening (“induration”) on the treated side. Importantly, these genes were not observed in the 2-week treated samples or the 4-week untreated group.^10^

In addition, further validation of molecular changes occurring within the dermis was provided by histological confirmation of increased collagen and elastin formation on the treated side, less edema was confirmed by ultrasound analyses, and decreased skin induration was demonstrated by fibrometer readings.^11^

In this situation, it was demonstrated that in patients undergoing body contour procedures, a topical treatment with the appropriate actives induced an accelerated healing response that involved the clearance of “waste” products (Figure 1) and the induction of anti-inflammatory genes and ECM remodeling, which translated to less edema, skin induration, swelling, and patient discomfort.^10^ It is beyond the scope of this paper to go into comprehensive details on the components of each formulation—these are detailed in previous publications.^5,9,14,24^ However, the most relevant active components are listed in Table 1 together with a description of their modes of action.

**Table 1.** Examples of Some Major Active Constituents and Their Actions in the Product Formulations

Agent	Mode of Action
Tripeptide-1	Releases gelatinase, clears ECM, stimulates neocollagenesis and neoelastagenesis^5^
Hexapeptide-12	Elastin binding protein draws in elastin and optimizes fibroblast function—synergistic activity with tripeptide-1 on scar control (TGF-β3) and matrix remodeling^5^
Hexapeptide-11	Stimulates autophagy, aiding macrophages in lipid droplet and debris absorption and cellular recovery^10,25^
Acetyl tetrapeptide-2	Stimulates elastin formation through LOXL1 activity^10^
Phosphatidylserine	Aids in macrophage activity (“eat me” signaling), stimulates procollagen, improves glycation end product elimination^5,10,25^
Lactoferrin	Plasmin inhibitor—aids in macrophage activity optimization, biofilm inhibition^25^
Oleuropein	Anti-inflammatory, antimicrobial, stimulates ubiquitin and autophagic processes^5^
Phytoene/phytofluene	Colorless carotenoids derived from saltwater microalgae that modulate prostaglandin E2 (PGE-2) and have the ability to quench oxygen radicals^25^
Naringenin	Citrus/vitamin B derivative, antioxidant, anti-inflammatory, reduces erythema and irritation^5^
Tetrandrine	Inhibitor of smad 2/3 signaling, increase smad 7—scar control^26^
*Centella asiatica*	Inhibitor of smad 2/3 signaling, increase smad 7 and TGF-β3^27^
Xylitol	Antibiofilm synergistic activity with lactoferrin^25^

ECM, extracellular matrix; LOXL1, lysyl oxidase-like enzyme 1; smads, intracellular proteins that transduce extracellular signals from TGF ligands to the nucleus where they activate downstream gene transcription; TGF-β3, transforming growth factor beta 3.

These findings introduce a new concept of skin bed preparation and treatment in conjunction with surgical procedures in an effort to influence the healing trajectory and improve outcomes and patient experience. From a practical standpoint, the case for preconditioning resides in the changes that take place within the ECM, resulting in improved crosstalk and signaling between cells (primarily fibroblasts) and ECM proteins. Optimization of this crosstalk ensures that the healing profile can begin as soon as needed and hastens the process of inflammation resolution and the initiation of regeneration.^14^

Previous studies, including our study noted above, confirm that together with TNF-α and IL-1, IL-6 is considered a major proinflammatory cytokine important in the early control of the healing process. IL-6 is an important modulator of CD4 T-cell effector functions, shaping the immune response and contributing to inflammation.^27^ In addition, as demonstrated in our gene study above and in common with other studies, we show that IL-6 modulated the Th1/Th2 balance towards a Th2 profile.^10,27^ IL-6 also promotes IL-4 production, which further enhances Th2 differentiation through an autofeedback loop.^27^

IL-6 expression is subject to both basal homeostatic regulation and rapid induction in the context of stress, such as surgery.^28^ When released by neutrophils, IL-6 signals a potential danger response affecting innate and adaptive immunological outcomes. IL-6 promotes macrophage differentiation and also inhibits the activation of the transcription factor NF-κB promoting an alternatively activated macrophage phenotype associated with wound healing. Hence, IL-6 has clear proinflammatory effects, but these effects are temporal, and if the milieu is conducive, IL-6 then also coordinates the anti-inflammatory activities essential for the resolution of inflammation.^28^ Dysregulation of IL-6 signaling can lead to either fibrosis or a failure to heal.^29^ As inflammation progresses, IL-6 signaling is responsible for the switch to a reparative environment. The regulation of wound healing is critical: inappropriate proinflammatory signaling can result in wounds that take much longer to heal, and if the switch to proliferative signaling is not carefully controlled, fibrosis may result from the excessive accumulation of ECM proteins, such as collagen, at the surgical site.^29^

Further delving into the subject, the impact of the acute inflammatory response induced by liposuction differs from that of chronic inflammation. Study results indicate that liposuction causes only a transient elevation of acute inflammatory markers, such as IL-6, high sensitive C-reactive protein (hCRP), and serum amyloid A (SAA), and a transient decrease of NO. The impact of liposuction for normal subjects did not advance beyond acute inflammatory response.^30^ This study showed that only IL-6, hCRP, and SAA responded acutely to the insult and became elevated 1 day following liposuction. However, both proinflammatory cytokine and acute phase reactants returned to the baseline 1 month following the surgery.^30^ Our series shows that proinflammatory markers may have decreased earlier at the 2- to 4-week interval. In addition, liposuction transiently raised and reduced the level of leptin and adiponectin, respectively.^30^

Thus, acute phase reactors and inflammatory mediators may be influenced by the application of a topical preparation. In this context, the macrophages in the wound microenvironment also play critical roles during the healing process. M1 macrophages engulf necrotic/apoptotic neutrophils, waste products, or damaged cells and produce proinflammatory cytokines, such as IL-1β, IL-6, and TNF-α, which amplify the inflammatory response in the early stages of wound healing.^31^ In the later stages of healing, M2 macrophages produce anti-inflammatory cytokines and growth factors, such as IL-10, TGF-β1, PDGF, VEGF-A, FGF2, and TGF-α. These macrophages play pivotal roles in downregulating inflammation and promoting healing by stimulating the proliferation of keratinocytes, fibroblasts, and endothelial cells, encouraging angiogenesis, and synthesizing ECM molecules to restore the damaged tissue.^31^

As previously noted, adipose tissue, due to its anatomic proximity and relatively large tissue volume, is traumatized in surgical procedures. Clinical links between adipose tissue biology and surgical outcomes are emerging.^32^ For instance, exacerbated adipose tissue IL-6 release in obese surgical patients correlates with perioperative insulin resistance. Additionally, surgical trauma upregulates markers of inflammation, matrix remodeling, and angiogenesis, and preoperative diet can modulate this response.^32,33^

In this context, the macrophages may benefit traumatized tissue by clearing cellular debris and preventing further activation of the immune system. Due to its plasticity and its ability to modulate the inflammatory status by the secretion of pro- and anti-inflammatory adipokines, adipose tissue stands as a prime interventional target.

## DISCUSSION AND CONCLUSION

The concept of preparing the patient for surgery prior to the procedure with the hope of improving outcomes is not new. This period offers a window of opportunity to optimize the patients’ nutritional, functional, and psychological state prior to surgery.^34^ Traditionally, these efforts have concentrated on “fitness for surgery” and avoiding potentially harmful agents. In addition, there is evidence supporting preoperative exercise training, dietary restrictions, smoking cessation, reduction in alcohol intake, anemia management, and psychosocial support.^33,34^ A focus that has not received much attention is the local treatment of the operative area. Aside from the attempted sterilization of the surgical site with antiseptic preparations to combat surgical site infections, no concerted effort has been made to try to influence healing outcomes by applying topical agents to the skin.

Recent advances in peptide technology together with synergistic chemical compounds have created possibilities for clearing dysfunctional ECMs and replacing them with a milieu more conducive to regenerative healing. This likely occurs due to the improved “crosstalk” within the ECM that allows for improved cellular function, increased molecular signaling, and protein production in relation to the topical stimulation.^5,14^ Evidence of the efficacy of this approach has been demonstrated in clinical trials studying surgical preconditioning,^14^ facial resurfacing,^35,36^ and recently in invasive surgical procedures.^10-13^ In addition, the possibility of a topical preparation affecting structures within the subcutaneous tissue through liposome delivery systems to improve macrophage efficiency and autophagic stimulation^9,10,37^ has introduced mechanisms for a hastened recovery, improved patient experience, and optimized outcomes to invasive surgical procedures. These avenues have provided a new paradigm to the perisurgical space, that of topical dermal treatment influencing surgical outcomes. With further research, including multicenter clinical trials that are underway with a single product incorporating the applicable actives for preconditioning and postsurgical healing, new and exciting possibilities may emerge.
